# Nanomaterial Adjuvants for Veterinary Vaccines: Mechanisms and Applications

**DOI:** 10.34133/research.0761

**Published:** 2025-07-08

**Authors:** Li He, Ruliang Pan, Rui Liang, Bingyao Li, Pei Zhang, Shujun He, Baoguo Li, Yuli Li

**Affiliations:** ^1^ Shaanxi Key Laboratory for Animal Conservation, College of Life Science, Northwest University, Xi’an 710069, China.; ^2^ International Centre of Biodiversity and Primate Conservation, Dali University, Dali 671003, China.; ^3^ School of Human Science, The University of Western Australia, Perth, WA 6009, Australia.; ^4^ Shaanxi Institute of Zoology, Xi’an 710032, China.; ^5^ College of Life Science, Yanan University, Yanan 716000, China.

## Abstract

Safe and effective veterinary vaccines prevent infectious diseases and reduce morbidity. In this field, nanovaccines based on nanomaterials are emerging, showing great potential as innovative alternatives to conventional vaccines. This paper highlights the advantages, disadvantages, and mechanisms of nanomaterials, including biomimetic, polymeric, lipid nanoparticles, self-assembling proteins, and other materials used in veterinary vaccine development. We also describe the progress of their research in developing vaccines against common and serious veterinary infectious diseases, such as foot-and-mouth illness, porcine epidemic diarrhea, pseudorabies, and bordetellosis. We aim to provide a scientific basis and practical guidance for the research and development of new veterinary vaccines, thereby contributing to scientific and technological progress in the field of veterinary medicine and the protection of animal health.

## Introduction

The diseases caused by the spread of infectious pathogens have become increasingly prominent and severe. This not only substantially affects the productivity of the livestock industry and economic development but also poses a serious threat to public health [1–3]. Therefore, it is necessary to improve the prevention measures of animal diseases. This can be achieved through vaccination, the most effective and economical means of combating infectious diseases. However, conventional vaccines have many limitations in their application. For example, attenuated vaccines have the risk of reversion to virulence and require high storage and transportation conditions; inactivated vaccines have a short immunity period and require multiple vaccinations; subunit vaccines have poor immunogenicity due to the lack of pathogen-related molecular patterns; RNA vaccines are unstable and require harsh storage conditions; and DNA vaccines do not stimulate strong immune responses [4–6]. At the same time, infectious pathogens continue to evolve and may even reach pandemic levels, and existing vaccines may not adequately meet the actual needs [7,8]. Thus, the development of veterinary vaccines has a long way to go and requires continuous in-depth research and innovation.

Fortunately, nanomaterials offer new opportunities and hope for the development of veterinary vaccines. They are materials with at least one dimension of their structure in 3 dimensions at the nanoscale (1 to 100 nm) or are composed of nanostructured units with remarkable properties. Because of their unique nanoscale effect, nanomaterials can augment the immunogenicity of antigens, promote the antigen-presenting cells (APCs) to take up antigens, regulate the release of antigens, and thus effectively activate immune responses [9–11]. At the same time, nanomaterials can potentially develop multivalent vaccines [9]. In addition, they also enrich the vaccination route, improve transdermal immunity, and optimize the effect of mucosal immunity, especially against respiratory viruses, to ensure the better health of livestock [11].

In this review, we first address the benefits of nanomaterials in veterinary vaccine development. Then, we address the roles, advantages, disadvantages, and optimization strategies of various nanomaterials in veterinary vaccine development. Because nanomaterials are often used in combination with multiple materials in the actual development of veterinary vaccines, understanding the properties of each material is a prerequisite for the rational use of nanomaterials. Finally, the research on nanomaterials for vaccines against highly prevalent and dangerous infectious diseases in the past 2 years is highlighted to supply references for developing new veterinary vaccines.

## Advantages of Nanomaterials for Veterinary Vaccine Development

The components of a vaccine consist mainly of an antigen and an adjuvant. Since the antigen is less immunogenic, an adjuvant is added to strengthen the immune response to the target antigen [12]. Adjuvants can be categorized into vaccine delivery systems and immunostimulants. Nanomaterials can function as adjuvants to enhance the immune response in vaccines, mainly by stimulating the immune system, and have many advantages over conventional adjuvants [12–15], especially in the development of innovative veterinary vaccines [16].

### Enhancing antigen immunogenicity and stability

Conventional inactivated and subunit vaccines are safe but weakly immunogenic [17]. The nanoscale size makes nanomaterials close to the size of natural viruses, and the large surface area allows them to encapsulate antigens at high densities or to distribute antigens in a highly ordered manner on their surfaces, facilitating their recognition by the immune system and enhancing antigenic immunogenicity [18]. Self-assembled nanoparticles (NPs) serve as platforms for displaying homologous or heterologous antigens, maintaining high antigenic density and repetitive antigenic display, and have been utilized for the antigenic display of severe acute respiratory syndrome coronavirus 2 (SARS-CoV-2), influenza viruses, avian influenza viruses, and rabbit hemorrhagic disease viruses, among others [19–21]. In addition, mosaic NP vaccines with heterotypic antigens have broad-spectrum antiviral capabilities, and the spatial location and proportion of the heterotypic antigens are essential for the strength of vaccine-induced immune responses. Zhang et al. [22] utilized the heterotypic antigen spatial assembly strategy of DNA nanotechnology to guide the design of mosaic NP vaccines. It was shown that equal proportions of heterotypic antigens produced more broad-spectrum neutralizing antibodies than unipolar and bipolar distributions.

Stability is a key factor in vaccine efficacy. Protein and nucleic acid antigens can change their structure due to changes in temperature and pH, affecting the immunization effect of the vaccine, especially nucleic acid vaccines, where less than 1% of the injected dose of nucleic acid can be delivered to the target cells in an active form under routine injection [23]. In addition, the adjuvant’s stability also affects the vaccine’s overall effectiveness. For example, when agglomeration or precipitation occurs in traditional aluminum salt adjuvants, they cannot function properly to adsorb antigens and stimulate immune cells, reducing the vaccine’s effectiveness. Nanomaterials offer new strategies to improve the stability of vaccines. Some nanomaterials, including metal NPs (MeNPs) and metal-organic frameworks (MOFs), inherently possess good stability and can protect antigens from environmental factors after encapsulating them. For example, the vaccine of *Pseudomonas aeruginosa* prepared by Chen et al. [24] with palmitic-acid-modified MOFs has good thermal stability. In addition, lyophilization technology can enhance the stability of messenger-RNA-lipid NPs (mRNA-LNPs). Li et al. [25] developed a highly efficient lyophilization method for mRNA-LNPs by incorporating a mixture of lyophilization protectants, including mannitol, alginate, and sucrose, which reduced the lyophilization process duration from 40 to 100 h to 8 to 18 h. They significantly reduced the production cost, and the lyophilized mRNA-LNPs exhibited good thermal stability. The lyophilized mRNA-LNPs are stabilized at 2 to 8 °C, while the immunogenicity of the antigen remains unchanged.

### Improving access to vaccination

Vaccination strategy affects the intensity and type of immune response a vaccine elicits. Although traditional vaccination is somewhat effective, the optimal vaccination strategy varies among vaccines, and improper immunization may result in poor vaccine outcomes. Most traditional vaccines are administered via injection, but injectable vaccinations typically require strict cold chain storage and specialized inoculation by medical personnel, can be traumatic and painful for the animal, and often result in mucosal immunodeficiency [26]. The development of nanomaterials provides multiple options for vaccine routes, including oral, intranasal, and immersion administration for infectious disease treatment.

#### Oral vaccination

Oral vaccination of animals has several notable advantages, as the vaccine can be administered naturally by simply mixing it into the feed or adding it to the drinking water [27]. This considerably reduces the difficulty and workload of vaccination, making it particularly suitable for large-scale breeding groups [28]. Moreover, oral vaccines can not only elicit a systemic immune response but also activate mucosal immunity. However, few oral veterinary vaccines have been developed [29]. The main reason is that antigens are broken down by enzymes in the gastrointestinal tract and lose their immunity, making it difficult for them to play an immune role. Nanomaterials can serve as a delivery system to protect the antigen across the gastrointestinal tract, penetrate intestinal mucus, be absorbed by intestinal cells, and then enter the bloodstream through either the mesenteric vein or the lymphatic vessels. In the lymphatic route, the vaccine is transported from the enterocytes to the mesenteric lymphatics and finally enters the bloodstream through the thoracic duct, improving oral vaccine bioavailability. Zhao et al. [30] developed *N*-2-hydroxypropyl trimethyl ammonium chloride chitosan (CS)/*N*,*O*-carboxymethyl CS NPs (SA@N-2-HACC/CMCS NPs) acidified and encapsulated by aluminum sulfate sucrose complexes and using bovine serum albumin as an antigen, which had a retention time of more than 12 h in the intestine after oral administration and triggered the production of secretory immunoglobulin A (sIgA) and IgG.

#### Intranasal administration

Compared with oral vaccines, intranasal vaccines have a lower potential for antigenic degradation due to lower enzyme activity in the nasal cavity than in the gastrointestinal tract [31]. Moreover, intranasal vaccination is an ideal mode of vaccination for respiratory viruses, as it can elicit an immune response directly at the site of viral invasion and effectively prevent viral infection. However, the ciliary clearance of nasal mucus influences the residence time of antigens within the nasal mucosa, making it difficult for antigens to adhere to the nasal mucosa over a prolonged period, resulting in limited capture of antigens by APCs in the nasal epithelium [32]. Fortunately, some nanomaterials can prolong the dwell time of the antigen within the nasal cavity and enhance delivery efficiency. Moreover, NPs technology enhances immune activation by generating higher protection and antibody titers [33]. Liu et al. [34] utilized a charge-assisted stabilization (CAS) strategy to enhance the LNPs’ stability and developed intranasally inhaled CAS-LNP vaccines that achieved efficient pulmonary mRNA delivery in mice, dogs, and pigs, triggered strong mucosal and systemic immune responses, and created the basis for the development of intranasal inhaled mRNA vaccines with an excellent potential for pulmonary infectious diseases—the intranasal vaccine with mannose-CS NPs designed by Bugybayeva et al. [35] can induce sIgA antibodies in the respiratory tract at levels exceeding those caused by commercially available swine influenza A virus (IAV) vaccines. In conclusion, intranasal administration provides a powerful strategy for effectively combating respiratory infections.

#### Immersion

Immersion vaccination offers unique advantages for mass inoculation in aquaculture, which can be readily implemented by submerging fish in water containing the vaccine. This method is efficient, convenient, and demands fewer human resources [29]. However, the efficiency of immersion vaccination is low because of the multiple barriers of the skin and gill epithelium. Nanomaterials with mucosal adhesion properties and intense penetration have been developed as carriers to overcome these barriers, such as CS and carbon nanotubes (CNTs). For example, Kitiyodom et al. [36] utilized a CS composite nanovaccine (CS-NE) to prevent *Flavobacterium columnare* infection. After immersion vaccination, the relative survival rate of the vaccinated experimental fish group was 78%. In contrast, the mortality rate of the unvaccinated control group of fish reached 89%. Compared with the whole-cell-vaccinated group of fish and the control group, the mucosal epithelium of the CS-NE-vaccinated fish had a stronger ability to take up antigens. Gene expressions, such as IgM and tumor necrosis factor-α, were significantly up-regulated in fish gills.

### Targeting the lymph nodes and activating APCs

Vaccines can reach lymph nodes (LNs) and activate APCs through 2 main pathways. One is that after APCs capture and internalize antigens, the pattern recognition receptors (PRRs) of APCs identify pathogen-associated molecular patterns (PAMPs) or damage-associated molecular patterns (DAMPs), activate downstream signaling pathways, cause the up-regulation of major histocompatibility complexes (MHCs) and costimulatory molecule expression, lead to APCs maturation, and migrate to LNs [37]. In LNs, APCs process and present antigens to adaptive immune cells, thereby initiating adaptive immune responses [38]. Some nanomaterials can promote APC activation during the APC activation process by activating nucleotide-binding oligomerization domain-like receptor protein 3 (NLRP3) inflammasomes, initiating Toll-like receptor (TLR)-dependent pathways, complement pathways, etc. [39,40]. For example, polyanhydride NPs promote the release of T helper 1 (T_H_1)-type cytokines through multiple TLR pathways [39]; poly(γ-glutamate) NPs promote dendritic cell (DC) maturation through TLR4 and myeloid differentiation marker 88 (MyD88) signaling pathways, activating potent innate and adaptive immune responses [41]. Moreover, nanomaterials can simultaneously deliver antigens and PRR agonists to further enhance APC activation [42]. Li et al. [43] has designed a universal, purely biological nanovaccine system consisting of 3 modules: stimulator of interferon gene (STING) agonists, self-assembled NPs, and delivery vectors targeting the cell membrane surveillance system. This system exhibits excellent LN targeting and broad-spectrum antiviral efficacy, making it a highly diversified and potent vaccine platform.

The other pathway relies on passive diffusion through afferent lymphatic vessels to reach LNs and then be captured and activated by APCs within the LNs. Still, in this pathway, the antigen rapidly enters capillaries rather than lymphatics, which usually accumulates in peripheral tissues [44]. However, peripheral tissues contain only a small number of immune cells, which are insufficient to stimulate a strong immune response against infectious diseases. Therefore, it is crucial to develop delivery systems that effectively target antigens to the LN and activate an immune response. Some nanodelivery systems can naturally migrate to the LN due to their unique size characteristics, with particle size being a key factor; nanomaterials with diameters of 10 to 100 nm can naturally reach the LN through lymphatic vessels, while nanomaterials with diameters greater than 100 nm usually need to be internalized by DCs before they can be transported to the LN [45]. Guo et al. [46] demonstrated that mesoporous silica NPs (MSNs) with smaller particle sizes exhibit a more potent lymphatic targeting efficiency. Besides particle size, other features of nanomaterials, such as charge, surface modification, and hydrophilicity, are also critical for their accumulation in the LN [47]. It was found that polyethylene glycolization increased the transport efficiency of 40- and 100-nm NPs across lymphatic endothelial cells by 50-fold compared to unmodified NPs and that the transport efficiency was maximized when the polyethylene glycol had a high grafting density or was in a dense-brush-like conformation and did not vary depending on the size of the NPs [48]. Interestingly, Wu et al. [49] developed a complex emulsion (W_NP_/O/W) with strong deformability, where the internal aqueous phase utilizes CS NPs for efficient antigen loading. The internal positively charged particles, endowed with a flexible oil layer, achieve excellent deformability, which enables LN-targeted delivery and sustained antigen enrichment. In addition, nanomaterials, when functionalized with ligands such as small molecules, peptides, or antibodies, can achieve active targeting of subpopulations of APCs, bind specifically to receptors on target cells, enhance antigen accumulation at the target site, and reduce damage to other nontarget tissues and organs. For example, Vu et al. [50] used ferritin NPs conjugated with anti-C-type lectin receptor family 9 member A antibodies to achieve targeting of DCs in the LN, resulting in concentrated antigen deposition within the germinal centers and triggering a strong antibody response.

### Controlling release

Developing vaccine delivery systems with controlled release kinetics has long been a challenge. The degradation rate of poly(lactic-*co*-glycolic) acid (PLGA) can be regulated by varying the ratio of lactic acid to glycolic acid, molecular weight, etc., and the vaccines encapsulated in them are slowly released as the PLGA is gradually degraded [27]. Some nanomaterials are responsive to stimuli (temperature, pH, alternating magnetic fields, and enzymes) and can precisely control the release of antigens, such as nanogels (NGs). In addition, nanovaccines embodied MSN, and *Streptococcus agalactiae* antigens exhibit pH release properties, protecting against antigens in a gastric fluid environment at pH 1.5 and releasing antigens in an intestinal climate at pH 7.4 [51]. This responsive release design is also another level of control over cellular targeting, which can only be achieved when the nanomaterials are delivered to multiple cell types but only when released or expressed in specific cell populations due to the unique intracellular microenvironment that triggers a change in the properties of the nanomaterials that results in the release of the antigen. In addition, nanomaterials can be engineered to exhibit slow-release properties, as demonstrated by Zhang et al. [52], which delivered subunit vaccines using ovalbumin (OVA)@magnetic NPs encapsulated in gelatin methacryloyl microspheres, capable of sustained antigen release; Mayer et al. [53] achieved sustained release of antigens by loading an aqueous solution containing the target antigens into a lyophilized microporous annealed particle to create an antigen delivery platform, which forms a porous scaffold region that is instantly loaded with antigen and has slow-release properties.

Since a single dose of vaccine may not elicit an adequate immune response, vaccination often requires multiple doses. There is an urgent desire to develop a novel nanovaccine capable of sustained antigen release to maintain a long-lasting immune response with a single dose of vaccination but achieving long-term preservation and sustained antigen release in vivo is a challenging task. Wan et al. [54] encapsulated a rabies virus mRNA vaccine with lipopolymer complex NPs featuring a core–shell structure. A single low dose of this vaccine in mice triggered a strong humoral immune response and provided complete protection. Büyükbayraktar et al. [55] encapsulated the antigenic peptide epitope of *Mycobacterium tuberculosis* early secretory antigenic target protein with PLGA NPs. They utilized quaternized poly(4-vinylpyridine) to encapsulate the NPs, thereby achieving pulsed antigen release for up to 4 months. This indicates that nanomaterials are highly promising for developing single-dose vaccines. However, few single-dose veterinary vaccines have been developed, and further research is still needed.

### Promoting antigen cross-presentation

Endogenous antigens are delivered to CD8 T cells via MHC class I molecules. APCs such as DCs present exogenous antigens to MHC class I molecules via cross-presentation. Cross-presentation of antigens occurs either because antigens are internalized and escape from endosomes into the cytoplasm or because antigens are delivered directly to specific endosomes containing MHC class I molecules [56,57]. Antigen cross-presentation is crucial for activating antigen-specific CD8^+^ cytotoxic T lymphocyte (CTL) responses and is a vital modality for generating antiviral immunity. The properties of nanomaterials themselves are a factor influencing antigen cross-presentation. For example, MSNs with larger pores exhibit higher cross-presentation efficiency [58]. In addition, other methods that promote cross-presentation include the use of photosensitive materials to destroy endosomal membranes with light irradiation, the proton sponge effect, and membrane fusion. The proton sponge effect utilizes cationic polymers, such as polyethyleneimine (PEI) or lipid materials, that absorb large amounts of protons in acidic environments. This results in the rupture of endosomes due to swelling, allowing the release of antigens into the cytoplasm. On the other hand, membrane fusion occurs when the endosome membrane fuses with other membrane structures, allowing the antigen to enter directly into the cytoplasm or other organelles, which, in turn, participate in the antigen presentation process.

**Fig. 1.**
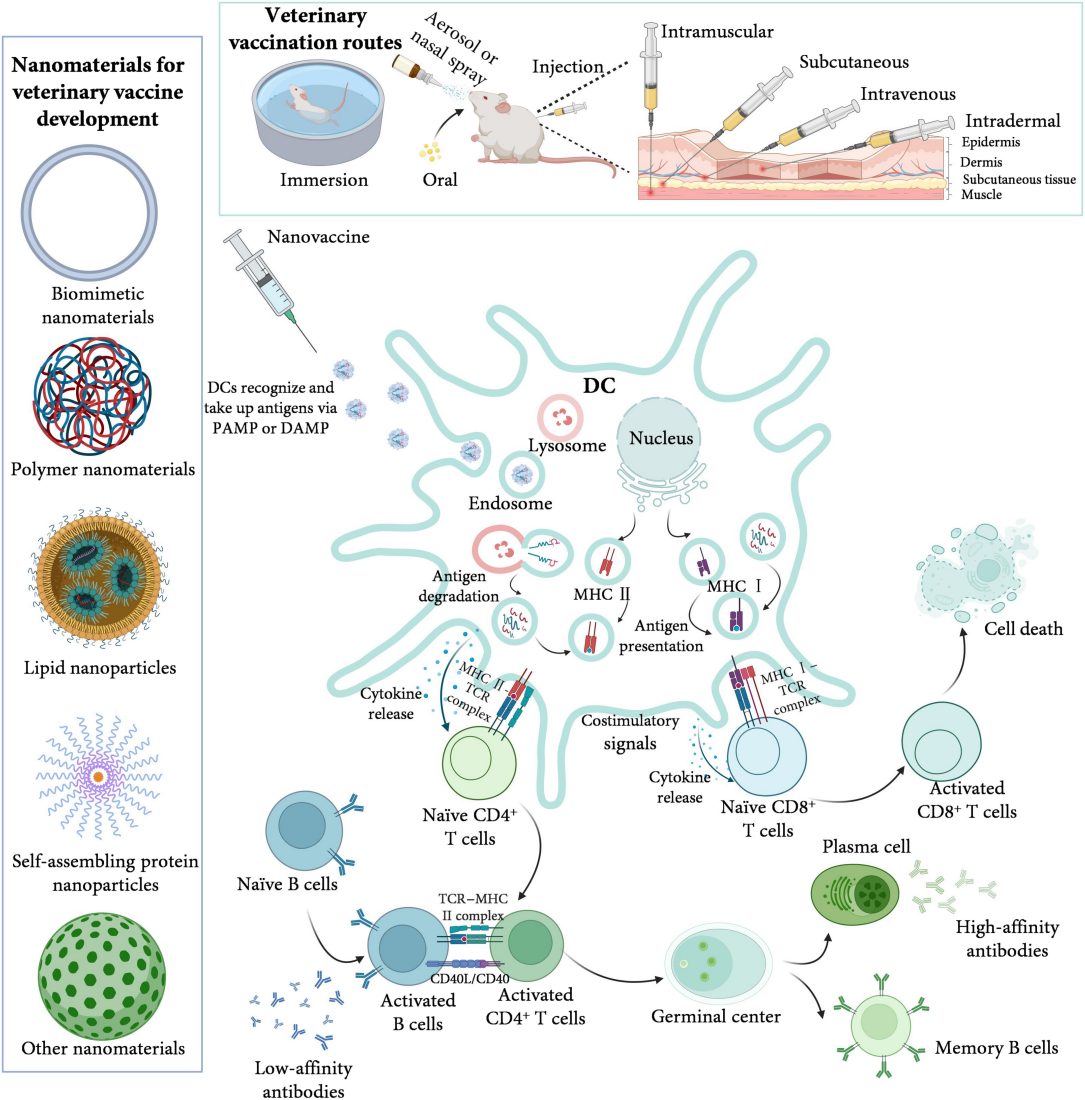
Vaccination routes, mechanisms of action of nanovaccines, and nanomaterials applied to veterinary vaccines. Nanomaterials used in the development of veterinary vaccines include biomimetic nanomaterials, polymer nanomaterials, LNPs, self-assembling protein NPs, and other nanomaterials. Veterinary nanovaccines can be inoculated through different routes, including injection, oral administration, nasal administration, and immersion immunization. Following vaccination, APCs, such as DCs, capture antigens through PAMPs or DAMPs, process the antigens, load epitope peptides onto MHC molecules, and then present them on the cell membrane to activate naive T cells. CD4^+^ T cells recognize MHC II–antigen–peptide complexes through T cell receptor (TCR) and release cytokines, stimulating B cells to differentiate into memory B cells and plasma cells. CD8^+^ T cells differentiate into effector T cells through MHC I–antigen–peptide binding and directly kill infected cells. Created with BioRender.com.

### Modulation of immune response type

DCs internalize exogenous antigens through endocytosis or phagocytosis, process into peptide fragments by proteases in endosomes, and present to CD4 T cells after binding to MHC II molecules to form complexes, which do not involve antigen cross-presentation [56]. Activated CD4 T cells differentiate into T follicular helper, T_H_17, T_H_2, and T_H_1 [59]. T_H_2 cells primarily activate B cells and activate humoral immune responses [60], whereas T_H_17 cells are pivotal in defending against extracellular bacterial and fungal infections and mediating inflammatory responses [61]. Different types of nanomaterials stimulate other types of immune responses. Moreover, the type of immune response induced by nanomaterials is influenced by various factors, including their nature, particle size, shape, and preparation method. Kumar et al. [62] used OVA as an antigen and spherical polystyrene particles to activate immune responses favoring the T_H_1 type. In contrast, rod-shaped particles induced immune responses favoring the T_H_2 type. Niosome NPs were prepared using the microfluidic mixing (MM) method and conventional film hydration (TFH) to develop influenza vaccines. The MM method produced carriers with a significantly homogeneous particle size distribution, which induced IgG1 antibodies and T_H_2-type responses. In contrast, the TFH method produced carriers with a higher dispersion of particle sizes, which caused a high level of IgG2a antibodies, interferon-γ (IFN-γ), and T_H_1-type responses [63].

Therefore, when using nanomaterials to develop veterinary vaccines, we must pay enough attention to the physicochemical properties of nanomaterials, including size and shape, surface charge and chemical composition, as well as hydrophobicity and hydrophilicity, and by regulating and controlling these properties, we can prepare nanoadjuvants with specific biological properties and develop ideal veterinary vaccines. At the same time, we also need to pay attention to the vaccination strategy and the ease of practical application, preferably to ensure the effectiveness of the veterinary vaccine while reducing the difficulty of the work. Finally, we also summarized the mechanism of action of nanovaccines (Fig. 1).

## Nanomaterials for Veterinary Vaccines

Adjuvants can improve the efficacy of veterinary vaccines. Still, they also have the potential to trigger harmful immune responses, and it is crucial to protect vaccine efficacy while reducing side effects. Therefore, the selection of nanomaterials is critical, and different nanomaterials play alternative roles in veterinary vaccines. Various nanomaterials are currently used as vaccine adjuvants, including biomimetic nanomaterials, self-assembled protein NPs,polymeric nanomaterials, and LNPs, and other nanomaterials.

### Biomimetic nanomaterials

Vaccines based on biomimetic nanomaterials attempt to improve vaccine efficacy and safety by replicating certain aspects of biology through synthetic or biosynthetic methods [64]. Biomimetic nanomaterials include virus-like particles (VLPs), cell-membrane-coated NPs (CNPs), and extracellular vesicles (EVs). The following section will describe each of these nanomaterials in veterinary vaccines.

#### Virus-like particles

**Fig. 2.**
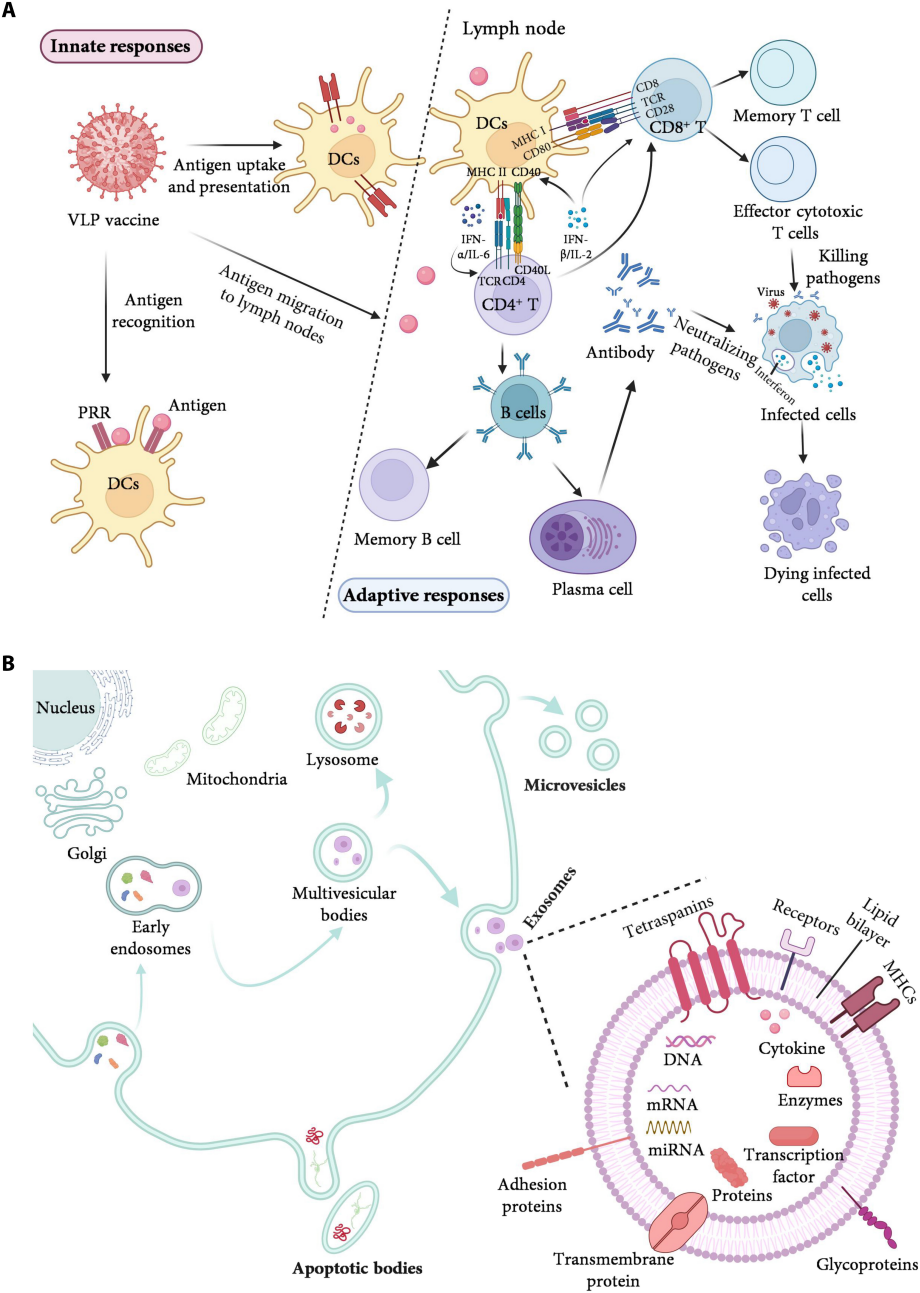
Biomimetic nanomaterials. (A) Mechanisms by which VLP-based vaccines activate innate and adaptive immunity. The immune system recognizes antigens carried by VLPs. DCs capture the antigens, process them, and form MHC–peptide complexes with TCRs on CD4^+^ and CD8^+^ T cells. CD4^+^ T cells activate B cells, which develop into memory B cells and plasma cells. Plasma cells release specific antibodies to eliminate pathogens. Activated CD8^+^ T cells develop into effector CTLs and memory CTLs. Effector CTLs initiate apoptosis of infected cells through the secretion of cytotoxic mediators. Created with BioRender.com. (B) Types of EVs, biogenesis pathways, and structural composition of exosomes. EVs can generate exosomes via inward budding from the inner membrane within the cell or microvesicles via outward budding from the cytoplasmic membrane. Apoptotic bodies are formed when the cell membrane invaginates and wraps around the cytoplasm during apoptosis. The biogenesis of exosomes: Endocytosis of the plasma membrane forms early endosomes, endosomes mature to form multivesicular bodies, multivesicular bodies contain luminal vesicles, and, subsequently, some multivesicular bodies merge with the cell membrane, releasing the luminal vesicles as exosomes outside the cell. Exosomes are rich in transmembrane proteins, glycoproteins, enzymes, transcription factors, mRNA, DNA, etc., which are involved in intercellular communication and regulate receptor cell function. miRNA, microRNA. Created with BioRender.com.

VLPs are highly structured protein particles with characteristics such as those of natural viruses, generated through the self-assembly of single or multiple structural proteins of viruses [65]. They are commonly used as a platform for vaccination. VLPs can be shown in a variety of expression systems, and VLPs self-assemble into a structure mirroring the original viral structure, with the target epitopes of the natural virus densely arranged on the surface and without any genetic material in the core, with high immunogenicity and safety [66]. Moreover, VLPs have virus-like permeability and retention ability, which can be effectively targeted to promote the effective release of antigen [67,68]. In addition, VLPs can be surface functionalized or coated with small molecules to improve circulating half-life and targeting specificity and optimize response properties to a given stimulus (temperature, pH, alternating magnetic field, and enzyme) [69,70]. Most importantly, VLPs have been shown to trigger both innate and adaptive immune responses (Fig. 2A).

Because of their high immunogenicity and ability to display multiple antigens, VLPs have been utilized to develop multivalent and single-dose vaccines. Single immunization with bivalent VLP vaccines can induce effective antibody immune responses, and all immunized poultry survived lethal challenges with H5N1 and H7N9 viruses, making them viable alternatives to traditional inactivated vaccines for preventing and controlling avian influenza virus infections [71]. However, VLPs are highly dependent on cold chain storage and transportation, and modified VLPs may be severely unstable because of the lack of viral genetic material. Therefore, special attention should be paid to its stability when modified VLPs are used. However, it is undeniable that the potential shown by VLPs far outweighs the current obstacles, and further research, mainly focusing on this aspect of scalable production, is necessary to market more and more vaccines against VLPs.

#### Cell-membrane-coated NPs

CNPs, consisting of a core of synthetic NPs encapsulated by cell membranes of natural origin, are a promising vaccine delivery system. CNPs combine the surface antigens and functions of source cells with the superior physicochemical properties of different NPs, providing advantages such as improved biocompatibility, reduced biotoxicity and immunogenicity, prolonged in vivo circulation time and half-life, and specific targeting [72]. Moreover, multiple types of cell membranes and NPs provide unique functions for CNPs, offering various options for vaccine development [73]. Most importantly, CNP-based vaccines have efficient lymphatic transport and delivery of antigens, but the stimulation of immune cells can be substantially enhanced by introducing additional payloads in the core of the NPs to deliver both antigens and immune enhancers or by introducing groups to modify the CNPs [74], such as Zhang et al. [75] encapsulating DNA vaccines encapsulated in mannose-modified scleractinian erythrocyte membranes with poly(d,l-lactide-*co*-glycolide) to construct a nanovaccine (PG@EM-M) against carp spring viremia virus infection. After vaccination, the efficiency of PG@EM-M uptake by APCs was significantly enhanced compared to that of the experimental group not modified by mannose, and a strong mucosal and systemic immune response was induced by intralesional gill inoculation. Similarly, the development of DNA nanovaccines against tilapia lake virus using mannose-modified erythrocyte-membrane-encapsulated CS as a delivery system (Cs-pS2@M-M) induced more antibody production, higher expression of immune-associated genes, and relative survival than the naked DNA vaccine and the same dose of non-mannose-modified Cs-pS2@M nanovaccine [76].

CNPs have made substantial research progress in the development of antimicrobial vaccines. Holay et al. [74] utilized macrophage-membrane-encapsulated NPs bound to anthrax toxin to develop an anthrax vaccine, producing long-lasting immunity with a single low-dose inoculation. However, the function of the single-cell membrane is both relatively challenging and straightforward in coping with the complex environment in vivo. In recent years, hybrid membranes obtained by mixing 2 or more cell membranes have appeared, but hybrid-membrane-encapsulated NPs have rarely been reported on veterinary vaccines, which can be strengthened for research applications in the future [77]. Moreover, no vaccine prepared with CNPs has been approved for marketing, and its safety remains a continuing concern. Scalable and optimized CNP preparation techniques should be developed to ensure reproducibility and reliability for large-scale production.

#### Extracellular vesicles

EVs refer to particles released by cells, encapsulated in a lipid bilayer, unable to self-replicate (without a functional cell nucleus). Almost all cells can produce EVs, and the composition of EVs from different sources has distinct differences [78]. According to their biogenesis, they are classified into 3 types: microvesicles, exosomes, and apoptotic bodies. The main ones used in vaccines are exosomes, which transfer biomolecules between cells through endocytosis, receptor-mediated capture, and fusion with the target cell membrane (Fig. 2B) [79]. The transfer of these molecules between cells triggers a functional response that mediates the immune response to pathogens [80]. In turn, the closed structure of EVs protects biomolecules and directs them to specific tissues based on the topology of membrane proteins [81]. Meanwhile, functionalized modification of the membrane surface of EVs using various biological, physical, and chemical means can confer unique targeting and functional properties [82–84]. Thus, EVs can play essential roles in immune processes, including antigen presentation, B cell and T cell activation, and inflammation [85].

EVs are widely available, have low immunogenicity, can be degraded in organisms, primarily through in vivo barriers, and are considered to be a new generation of delivery vectors with strong potential [86]. EVs have been utilized in the development of veterinary vaccines. For example, Zhu et al. [87] demonstrated that membrane vesicles (MVs) derived from the lipopolysaccharide-low-expressing avian pathogenic *Escherichia coli* (APEC) strain FY26Δ*msbB* could induce antibody responses in laying hens, promote bacterial clearance, exhibit cross-protective capacity, and effectively prevent infections caused by virulent APEC strains of serotypes O101, O78, O45, O7, and O1. *Actinobacillus pleuropneumoniae* (APP)-derived EVs (APP-EVs), a potential APP vaccine candidate, induced the maturation of DCs through a TLR4-dependent pathway, primarily causing a T_H_1-type IgG response, and were nontoxic, in contrast to commercial Coglapix vaccines, which induced a stronger T_H_2-type response but were notably toxic. Moreover, APP-EVs induced APP-specific T_H_17, T_H_1, and CTL responses and activated multifunctional T cells. Moreover, compared to Coglapix, APP-EVs improved the survival rate of APP-attacked mice, indicating a promising application prospect [88]. However, they have limitations such as complex surface modifications, a lack of standardized methods for high-throughput isolation and purification, and low yield [89]. In the future, we can attempt to prepare artificial vesicles that mimic natural EVs in composition and structure and utilize a standardized preparation process to achieve high-throughput production, thereby effectively addressing the issue of low yield associated with natural EVs.

### Polymeric nanomaterials

Polymer nanomaterials are extensively applied in vaccine development and other applications owing to their excellent biocompatibility, biodegradability, and ease of production [90,91]. In the following, we will introduce several polymer nanomaterials that show promising applications in vaccine development and elaborate on their unique properties.

#### Chitosan

**Fig. 3.**
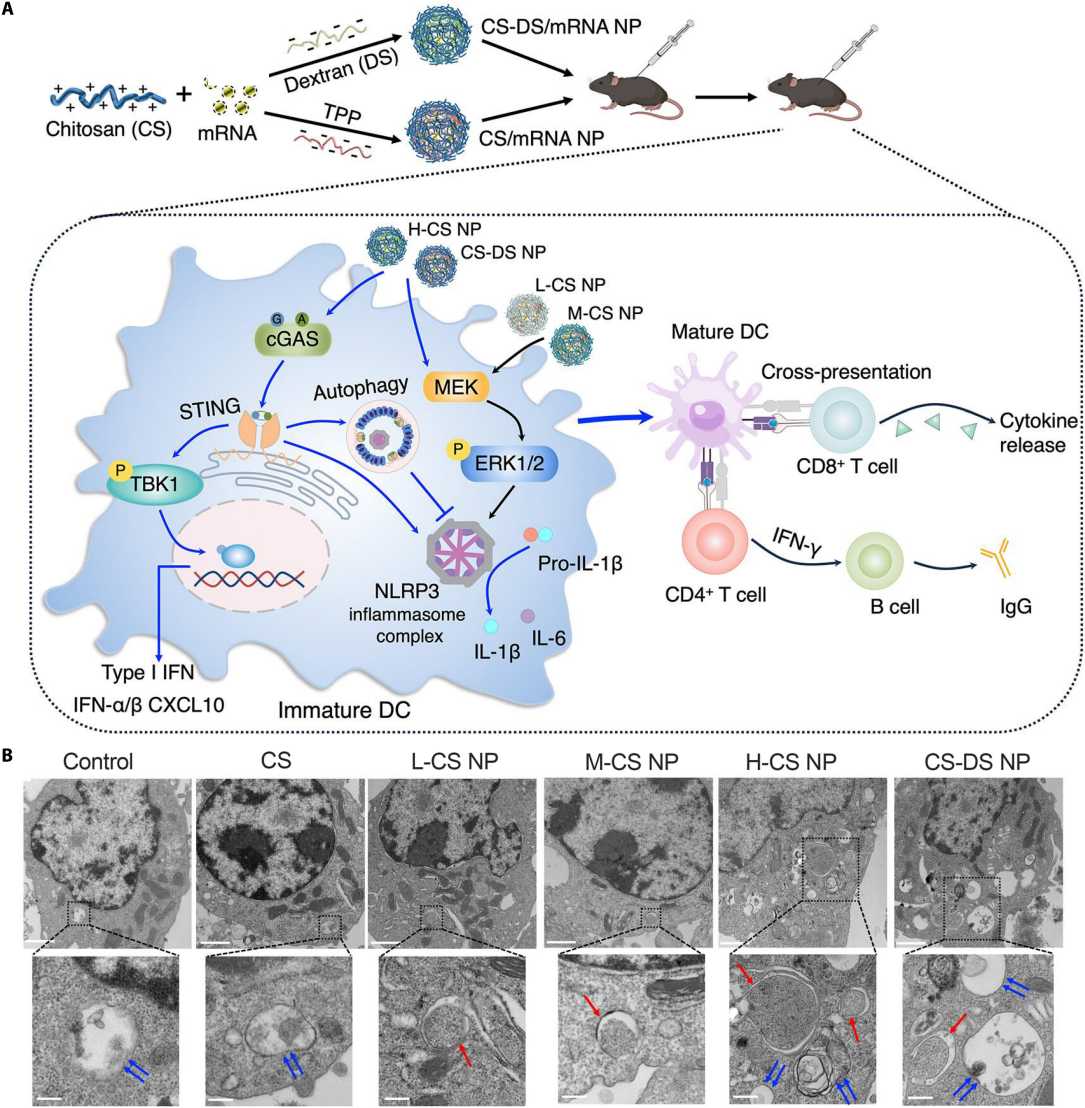
CS. (A) Schematic representation of the enhanced mRNA antigen-specific immune response to CS NPs of different molecular weights and its mechanism [94]. (B) Transmission electron microscopy images of cells exposed to CS NPs of different molecular weights [94].

CS, a deacetylated product of chitin, has been extensively explored as an adjuvant for vaccines because of its low toxicity, biocompatibility, biodegradability, adhesion, and enhanced biobarrier penetration [92]. CS not only promotes DC activation through the TLR-4-dependent signaling pathway but also induces type I interferon production and DC maturation by mediating cytoplasmic DNA activation of the cGAS-STING pathway, which enhances cellular immunity and produces large amounts of IgG2c [93]. Further, the role and mechanism of CS-NP-mediated immune enhancement when delivering mRNA vaccines are highly dependent on the molecular weight of the CS. Specifically, high-molecular-weight CS NPs initiate STING-mediated autophagy and NLRP3-related inflammatory vesicle signaling, which induces superior specific immune responses against mRNA antigens in vitro and in vivo. On the contrary, low-molecular-weight CS NPs induce only NLRP3 signaling and fail to trigger a strong immune response (Fig. 3) [94]. However, CS has limited solubility in most water and organic solvents [95]. Therefore, the surface of CS NPs needs to be chemically modified by introducing hydrophilic groups (hydroxyalkyl, carboxyalkyl, succinyl, thiol, catechol, etc.) or grafted polymers (polyethylene glycol, sodium alginate, etc.) to improve the solubility to overcome the in vivo environment [96–99]. For example, mannose CS NPs encapsulating *Salmonella* Enteritidis (SE) immunogenic outer membrane proteins (OMPs) and flagellin (FLA) can induce mucosal immunity through oral vaccination, which leads to a reduction in the amount of SE colonization in birds after exposure to the virus. Moreover, broiler chickens induced stronger cross-protection against *Salmonella* Typhimurium (ST). They produced more specific secreted IgY and IgA antibodies than the commercial Poulvac ST vaccine. Still, this vaccine reduced the load of the attacking ST bacteria in the cecum contents to a degree comparable to the Poulvac ST vaccine [100]. Ding et al. [101] developed a universal intranasal nanovaccine using thiolated CS to encapsulate VLPs displaying conserved T cell and B cell epitopes from the nucleoprotein and matrix 2 proteins of IAV. Following intranasal immunization, this nanovaccine provided complete protection against IAV strains from different host sources. CS and its derivatives have developed numerous oral, intranasal, and immersion vaccines against infectious diseases such as streptococcosis, columnaris disease, porcine circovirus type 2, *Mycoplasma hyorhinis*, *Mycoplasma hyopneumoniae*, *Aeromonas veronii*, and foot-and-mouth disease (FMD) [102–105].

In conclusion, CS has excellent potential in the field of veterinary vaccines. However, the complexity of the CS extraction process hinders the improvement of its purity and limits its biomedical applications.

#### Poly(lactic-*co*-glycolic) acid

PLGA is a polymer composed of hydroxyacetic acid and lactic acid, which are widely investigated polymers due to their biodegradability, biocompatibility, low immunogenicity, slow release, and nontoxicity for in vivo degradation [106]. PLGA NPs are mainly used as delivery systems for protein and nucleic acid vaccines. PLGA NPs not only encapsulate or load antigens to improve their stability but also increase uptake and cross-presentation by mimicking the shape and size of invading pathogens [107–110]. For example, the encapsulation of grass carp reovirus DNA vaccine and adjuvant with PLGA and polyvinyl alcohol nano-microspheres resulted in a 44% increase in protection rate compared to the control group, a relatively decrease in viral load, and a substantial enhancement in the expression of immune-related genes [111]. Meanwhile, by adjusting the ratio of lactic acid and hydroxyacetic acid in PLGA polymers, the degree of hydrophobicity can be regulated, which affects the rate of degradation and, consequently, the sustained and slow release of antigen [27]. However, the negative surface charge of PLGA NPs limits their interaction with negatively charged cell membranes and their intracellular uptake, and their mucosal adhesion and immune-enhancing ability are poor. Thus, PLGA modifications, including both binding to cations by covalent coupling or surface physical adsorption and hydrophilic modification, are needed to improve their physicochemical properties, making it an ideal delivery system [112].

Therefore, PLGA is a highly promising nanomaterial that has been used for loading antigens to develop vaccines against, among others, *Aeromonas hydrophila*, *Clostridium perfringens*, and Newcastle disease, with excellent immunization results [113–115]. However, the release rate of PLGA is challenging to regulate when controlling antigen release, and continuous research and innovation are necessary to optimize its performance. To compensate for this deficiency, PLGA can be used in combination with other nanomaterials that exhibit slow-release properties in the development of veterinary vaccines, or it can be encapsulated with cell membranes to form a core–shell structure. Excitingly, Neustrup et al. [116] developed a low-cost, easy-to-clean, reusable, modular microfluidic system for the preparation of PLGA NPs that can be loaded with proteins, encapsulated with efficiencies over 40%, and produced with a high degree of reproducibility, making them suitable for vaccine delivery.

#### Polyethyleneimine

The cationic polymer PEI, which consists of the vinylimine unit chain –CH_2_CH_2_NH–, offers the advantages of good water solubility and ease of synthesis and plays 2 leading roles in veterinary vaccines. One is a nucleic acid transfection agent to enhance the in vivo expression of the administered gene. PEI has a strong positive charge and maintains a considerable buffering capacity across a wide range of pH values. It facilitates its complexation with nucleic acids and release in vivo through the “proton sponge” effect [117]. However, PEI may show different cytotoxicity depending on molecular weight and structure and have a weak ability to recognize target cells, so modifications (chemical functional group modification, polyethylene glycol modification, linkage to oligosaccharides, targeting modification, fluorescent labeling, etc.) are needed to improve targeting and reduce toxicity [118–120]. For example, an *S. agalactiae* DNA vaccine using mannose-based polyethylenimine as a delivery system could produce higher serum antibody potency, induce higher expression of immune-related genes, and have a higher relative survival rate of 85.71% compared to other experimental groups [121]. Another approach is coating NPs to confer a positive charge [112]. NPs coated or modified with PEI can efficiently bind antigens, enhance antigen uptake by APCs, aid NP escape from lysosomes, activate macrophages and DCs, and induce T_H_1 immune responses [122,123]. For example, Zhang et al. [124] constructed a positively charged Pickering emulsion adjuvant system (PEI-CYP-PPAS) as an adjuvant for H9N2 avian influenza vaccine using PEI-modified yam polysaccharide PLGA NPs as stabilizers and squalene as the core. After immunization, PEI-CYP-PPAS induced higher hemagglutination inhibitory potency and IgG antibody levels than CYP-PPAS and aluminum adjuvant and promoted T cell activation, inducing cytokine expression.

In conclusion, PEI has multiple roles in vaccine development and is a critical material; however, its safety should be noted during the application process, and its dosage and biodistribution should be strictly controlled to minimize residue in animals.

#### Nanogel

NGs are NP hydrogels with 3-dimensional cross-linked polymer networks that integrate the characteristics of both hydrogels and NPs, safeguard their stability through chemical or physical cross-linking, are biocompatible and hydrophilic, have a swelling potential and a high specific surface area, and are responsive to a variety of stimuli (including biologics, pH, light, and temperature) [125–127]. NGs are mainly used as delivery vehicles for vaccines. They can load antigens and a variety of bioactive components to improve vaccine therapeutic efficacy and stability. Cationic cholesteryl-group-bearing pullulan (cCHP) NGs are extensively studied vehicles for nasal vaccine delivery. For example, intranasal immunization with formalin-inactivated *Staphylococcus aureus* coupled with cCHP NGs resulted in significantly elevated levels of anti-*S. aureus*-specific IgA antibodies in the milk of immunized ewes compared with unimmunized ewes, which inhibited the proliferation of *S. aureus* in the mammary glands of infected ewes [128]. Moreover, the size, surface modification, and chemical functionalization of NGs, as well as their stimulus responsiveness to various factors, can be adjusted according to the desired application. The specificity of targeted delivery can be enhanced by conjugating or surface-functionalizing NGs or compounds of NGs with biomolecules, such as proteins, ligands, or other molecules with molecular recognition specificity [127]. Finally, NGs can also be engineered to exhibit multiple antigens, allowing the production of multivalent vaccines that can defend against various viral strains or diverse infectious diseases [126].

Overall, NGs show great potential for enhancing the efficacy of veterinary vaccines, notably by preparing NGs with stimuli-responsive, tunable mechanical properties and possessing unique targeting properties for specific cell types and intracellular compartments. For example, thermoresponsive NGs based on poly(*N*-isopropylacrylamide) as carriers of APP outer membrane lipoprotein A (OmlA) antigens showed high biocompatibility in different cell lines, with anti-OmlA IgG titers comparable to those of conventional aluminum hydroxide adjuvant preparations and fluorescent signals detected predominantly in the lungs after intranasal administration [129]. However, before they can be widely used in clinical practice, the metabolic pathways, biodistribution, and possible immune responses of NGs in animals must be thoroughly investigated to determine their safety.

#### Dendrimers

Dendrimers are 3-dimensional nanostructures, hyperbranched macromolecules consisting of monomers that emanate radially from a central nucleus. They are highly molecularly homogeneous and size adjustable, have low immunogenicity and high surface functionality, and are highly water soluble, making them an excellent delivery system for vaccine development [130,131]. Biotargeting active compounds can be encapsulated in the voids of dendrimers, while nucleic acids, antibodies, and targeting peptides can be complexed or bound to the terminal surface groups. Overall, positively charged dendrimers may cause cytotoxicity, in contrast to anionic dendrimers, which are usually nontoxic [132]. Therefore, safety is a key concern when using dendrimers in the development of veterinary vaccines. In addition, the properties and performance of dendrimers can be tuned, and their relative toxicity may be reduced by adjusting the composition and number of terminal or branching moieties, modifying the central core, and introducing various functionalized portions on the surface [132,133]. The presence of multiple terminal functional groups makes dendrimers multivalent, enabling the development of multivalent veterinary vaccines. For example, when dendritic polylysine NPs were employed as adjuvants for developing H9N2 and H5N1 avian influenza vaccines, when adjuvants and antigens were compounded in a 1:3 ratio, higher levels of hemagglutination-inhibiting antibodies were induced compared with the bare antigens, a greater proportion of CD3^+^/CD4^+^ and CD3^+^/CD8^+^ T lymphocyte subsets, and cytokine production [134]. In addition, dendrimers have been investigated in the development of veterinary vaccines against FMD virus (FMDV), swine fever virus, rabies virus, and other pathogens [135].

The amphiphilic dendrimers, which have been studied over the past 2 years, combine the advantages of both lipid carriers and polymers [136]. Single-component ionizable amphiphilic Janus dendrimers (IAJDs) can be synthesized on a large scale and offer comparable advantages to 4-component LNPs used in commercial COVID-19 vaccines for targeted delivery of mRNAs [137–139]. We look forward to the future application of IAJD for veterinary vaccines.

### Lipid NPs

**Fig. 4.**
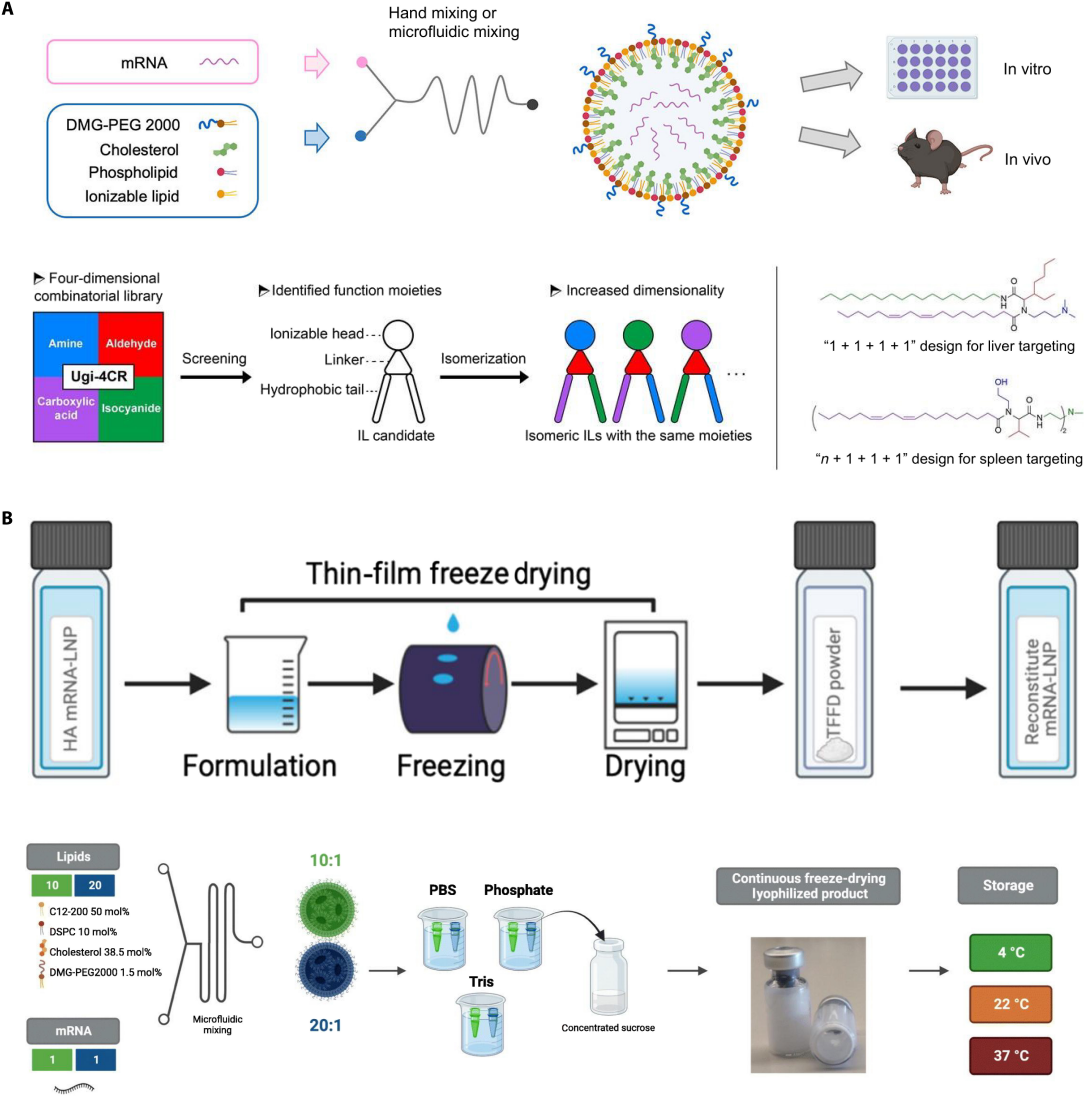
LNPs. (A) Establishment of ionizable lipid libraries by Ugi-4CR and MCR. Identification of ionizable lipids with the best performance in mRNA delivery by LNPs [143,144]. (B) Overcoming the cold chain with film freeze drying and continuous freeze drying of mRNA-LNP vaccines [145,146]. DMG-PEG 2000,1,2-dimyristoyl-rac-glycero-3-methoxypolyethylene glycol-2000; TFFD, thin-film freeze-drying; DSPC, 1,2-distearoyl-*sn*-glycero-3-phosphocholine; PBS, phosphate-buffered saline.

LNPs are extensively utilized as mRNA delivery systems, composed of polyethylene glycol, cholesterol, phospholipids, and ionizable lipids, which are coupled [140]. Surface modification is a viable strategy to boost the targeting ability of LNPs [141,142]. Besides modifying LNPs, modifying mRNAs and developing ionizable lipids can improve the targeting of mRNA-LNP vaccines, e.g., He et al. [143] and Isaac et al. [144] used the Ugi 4-component reaction (Ugi-4CR) and one-pot multicomponent reaction (MCR), respectively, to build ionizable lipid libraries to identify ionizable lipids that perform best in the delivery of mRNAs by LNPs (Fig. 4A). Moreover, mRNA-LNP vaccines can overcome “cold chain” transportation by lyophilization, membrane freeze drying, and continuous freeze drying (Fig. 4B) [145,146]. Moreover, certain LNPs possess intrinsic adjuvant activity, which relies on ionizable lipid components and the triggering of the interleukin-6 (IL-6) cytokine, rather than relying on MyD88 or mitochondrial antiviral signaling sensing of LNPs [147]. However, the adjuvant properties of LNPs vary depending on the lipid used and are usually weak and nonspecific. Because of the successful application of LNPs in the treatment of COVID-19, considerable research has been conducted on the development of veterinary vaccines using LNPs in recent years. Research has focused on the development of vaccines against avian influenza viruses [148–150], porcine deltacoronavirus [151], *Flavobacterium oreochromis* [152], *Chlamydia psittaci* [153], and other pathogens. In addition, Zhao et al. [154] utilized arginine-rich cationic LNPs as delivery vehicles for a DNA vaccine against *Echinococcus granulosus*, which was transfected in immune and nonimmune cells with a transfection efficiency nearly 2 orders of magnitude higher than that of a commercial reagent, and triggered a humoral immune response similar to that of the commercial adjuvant, as well as a significantly stronger cellular immune response, after intramuscular injection in mice. The vaccine has demonstrated great potential in combating zoonotic diseases, but subsequent infection testing is necessary to validate its efficacy.

Liposomes, the earliest LNPs, are critical vaccine delivery systems with the advantage of their flexibility to create numerous structures according to the chemical properties of the antigenic molecule and the sort of immune response desired [155]. Moreover, they can be chemically modified to target various cells and tissues in the organism [156]. In addition, by introducing pH-sensitive or heat-sensitive components, liposomes can release antigens in a controlled manner, but this may affect their stability and lead to premature release of antigens [156]. Compared to liposomes, solid LNPs (SLNs) and nanostructured lipid carriers (NLCs) are second-generation LNPs with higher stability [157]. In addition, NLC-based vaccines can be stored not only by transdermal immunization and nasal vaccination but also in the form of highly stable dry powder, which is very versatile and flexible [155]. The development of second-generation LNPs has substantially enhanced their performance for a broader range of applications. However, compared with NLCs, SLNs may have problems such as low antigenic loading and poor stability. The particle size distribution can be regulated to improve stability, or freeze-drying protectants can be added to reduce the structural collapse of SLNs during the freeze-drying process.

### Self-assembling protein NPs

Self-assembling protein nanoparticles (SAPNs) are formed by oligomerization of N-terminal pentameric and C-terminal trimeric convoluted helical sequence elements, which are biodegradable, biocompatible, highly immunogenic, and multivalent, and have great potential for veterinary vaccine development [158]. For example, Sun et al. [159] utilized thermophilic archaeal ferritin to generate 3 distinct SAPNs targeting epitopes of viral GP3, GP4, and GP5 proteins, respectively. These SAPNs were mixed to formulate a FeCocktail vaccine against porcine reproductive and respiratory syndrome. The vaccine effectively activated T cells in mice, protected piglets, reduced viral loads, and alleviated lung tissue damage. In addition, Chen et al. [160] employed *Helicobacter pylori* ferritin to develop an SAPN vaccine against H5N6 subtype highly pathogenic avian influenza virus, which induced potent antibody responses in chickens after a single immunization. The fused hemagglutinin (HA)–ferritin NP vaccine elicited significantly higher T_H_1/T_H_2 immune responses, conferred 100% challenge protection in chickens, and provided immune protection comparable to that of commercial vaccines, even at a low hemagglutination unit of 28. In addition to naturally occurring self-assembled proteins, vitro-assembled NPs enable the purification of antigenic proteins before assembly and provide better control over the quality of these proteins. For example, 2-component icosahedral protein NPs can be assembled in vitro to encapsulate multiple macromolecular cargoes, thereby combining the advantages of protein biologics with other therapeutic modalities, such as nonbiopolymers, small molecules, and nucleic acids [161].

Because of its multivalency and self-loading ability, SAPN is a safe and versatile vaccine platform that enables researchers to design novel vaccine candidates that trigger safe and long-lasting immune responses.

### Other nanomaterials

In addition to the nanomaterials as mentioned above, nanomaterials also include inorganic nanomaterials, carbon-based nanomaterials, and organic–inorganic hybrid nanomaterials [162]. Inorganic nanomaterials exhibit unique size-dependent structural, optical, electrical, and magnetic properties, which can be utilized for immunological applications by targeting various immune signals, enhancing stability, and delivering other insoluble cargos [163]. Carbon-based nanomaterials are used as vaccine delivery systems due to their ability to penetrate cells and their unique physical and chemical properties [164,165]. Organic–inorganic hybrid nanomaterials combine the advantages of organic and inorganic materials, offering controllable shape and size, as well as easily modifiable surfaces. MeNPs, CNTs, and MOFs are typical representatives of them, respectively. The following will introduce their roles in veterinary vaccines one by one.

#### Metal NPs

MeNPs are relatively nonbiodegradable, have a rigid structure, and are readily synthesized. They are immunostimulatory molecules that can generate both humoral and cytotoxic responses. Their immunostimulatory capacity correlates with the physicochemical properties of the NPs (e.g., size, charge, and hydrophobicity), and evidence suggests that they can aid in generating T_H_1 and T_H_17. They are also delivery systems that can enhance the immune response to pathogens, and at the same time, MeNPs have antimicrobial properties. MeNPs can migrate from the administration site, but careful toxicity monitoring is required. Gold NPs (AuNPs) and aluminum NPs (AlNPs) are MeNPs that have been used more frequently in veterinary vaccines [166]. For example, Xu et al. [167] used AuNPs and CS-modified *Viola philippica* polysaccharide NPs (CS-Au-VPP NPs) based on AuNPs and CS modification as adjuvants for porcine circovirus type 2 vaccine. After immunization, medium-dose CS-Au-VPP NPs significantly increased specific IgG antibody levels, T cell subset ratios, and cytokine contents. In addition, Liu et al. [168] utilized aluminum sulfate and N-2-hydroxypropyl trimethyl ammonium chloride CS NPs (N-2-HACC NPs) to prepare the nanoadjuvant N-2-HACC-Al NPs, which were employed as an adjuvant for developing a combined inactivated vaccine against H9N2 avian influenza and Newcastle disease. The vaccine induced higher-serum IgG, IFN-γ, and IL-4 levels than the commercially available combined inactivated vaccine, and its IFN-γ level reached more than twice that of the commercially available vaccine 7 d after immunization.

MeNPs have been employed in developing veterinary vaccines. However, some MeNPs are toxic and cause pollution when released into the environment. Finding a balance between safety, controllability, and environmental compatibility is a pressing issue that we must urgently address.

#### Carbon nanotubes

CNTs are one-dimensional quantum materials consisting of single or multiple layers of graphene curled around a central axis at a specific helix angle, categorized into multiwalled CNTs and single-walled CNTs (SWCNTs), which are utilized as delivery systems in vaccines [169]. CNTs exhibit low toxicity, strong adsorption and penetration capacities, excellent stability, and a lack of intrinsic immunogenicity [169,170]. Their special lumen can carry multiple antigens and safeguard them against degradation during delivery. At the same time, many ligands can be attached to CNTs, and, subsequently, the CNTs with attached ligands can deliver antigens to cells both in vitro and in vivo [170]. CNTs also have a large surface area, implying high surface reactivity and easy surface functionalization [171]. Moreover, CNTs are hydrophobic, and surface functionalization by encapsulation with biopolymers or covalent attachment of solubilizing groups to the outer wall and tip not only enhances water solubility and reduces toxicity but also highlights antigenic epitopes and is more conducive to the acquisition of specific antibodies through the targeted introduction of antigens to form stable antigenic complexes [171–173]. More importantly, vaccines based on functionalized CNTs can be administered by immersion, a convenient, stress-free method suitable for mass immunization. For example, functionalized CNTs were utilized as a delivery system to develop vaccines against *S. agalactiae* and *Streptococcus iniae* infections, resulting in a survival rate of more than 65% after immersion immunization in both cases [174,175]. Liu et al. [176] loaded peptides containing effector epitopes of neuronal necrosis virus onto SWCNTs to prepare a vaccine, which induced high antibody levels and up-regulated the expression of immune-related genes after immersion vaccination, resulting in a relative protection rate of more than 84.13% in fish. Moreover, not only CNTs can be modified, but also antigens can be modified to improve the targeting of the vaccine. For example, Zhao et al. [177] utilized functionalized, modified SWCNTs as the delivery system and mannose-modified major coat proteins as a targeted immunization vaccine against iridovirus disease. This vaccine resulted in the highest relative survival of 81.3% in mandarin fish after immunization, compared to 41.5% in the group without the major coat protein modification.

#### Metal-organic frameworks

**Fig. 5.**
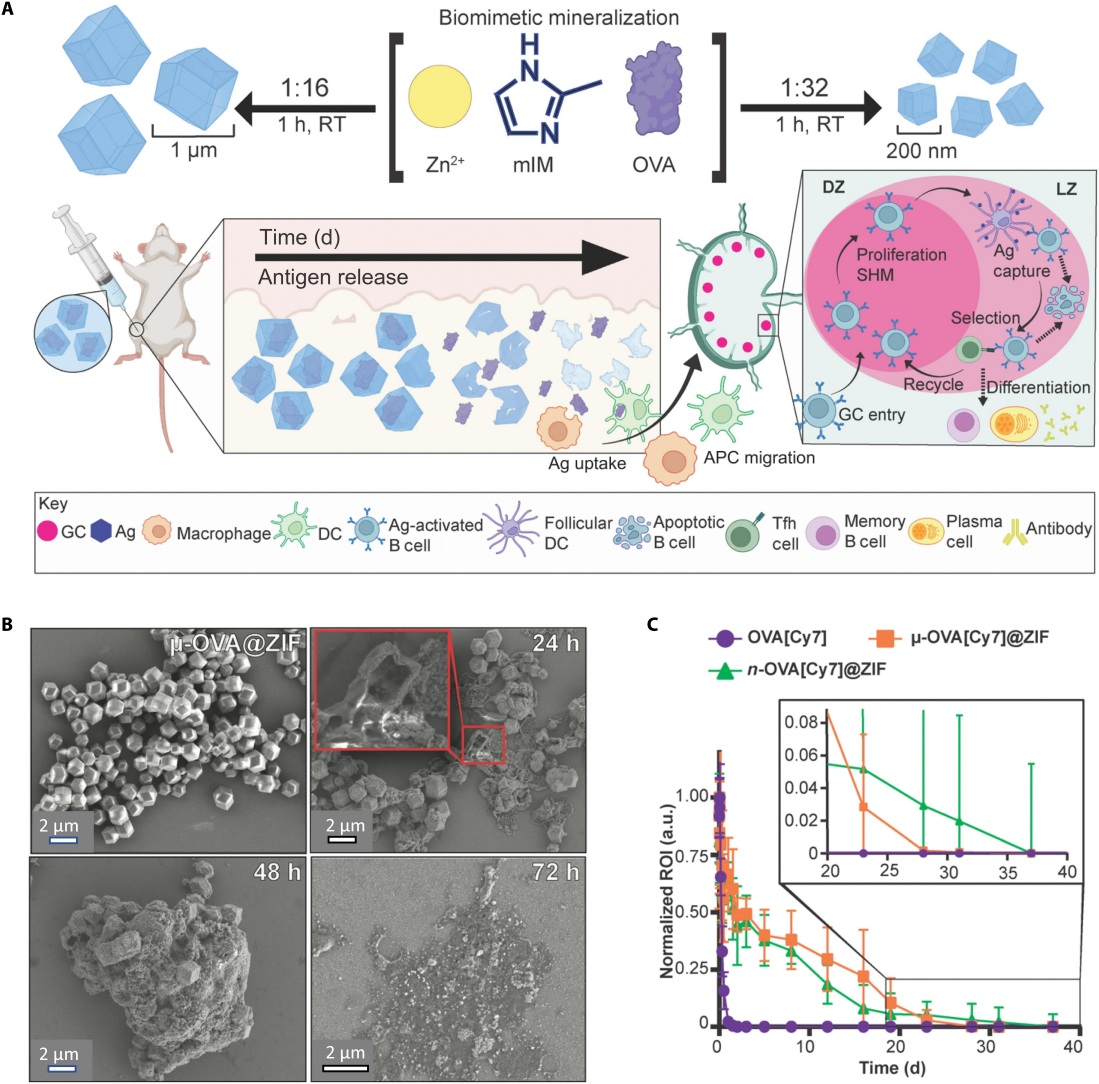
ZIF controls the sustained release of antigen. (A) Bionic mineralization of OVA@ZIF and sustained release of antigen [187]. (B) Scanning electron microscopy images of raw μ-OVA@ZIF and in vivo extracts at 24, 48, and 72 h after injection [187]. (C) Cy7 fluorescence in mice after subcutaneous injection of OVA@ZIF [187]. RT, room temperature; mIM, 2-methyl imidazole; SHM, somatic hypermutation; GC, germinal center; Tfh cell, T follicular helper cell; a.u., arbitrary units; ROI, region of interest.

MOFs are crystalline, porous materials with periodic network structures formed by the self-assembly of bridging organic ligands and metal clusters or ions. These possess the advantages of customizable dimensions and forms, as well as simple preparation and modification [178]. However, the poor stability of MOFs in aqueous media, mainly due to decomposition and coalescence under physiological conditions, severely restricts their application in the biomedical field [179,180]. Fortunately, suitable metal–ligand pairs can be selected to avoid the decomposition of MOFs. More importantly, different functional building blocks can be chosen or designed, or other classes of functional groups can be introduced through postmodification methods, allowing for targeted property modulation to prepare MOF materials for specific applications [181]. In addition, MOFs have been utilized as a strategy to overcome the cold chain. On the other hand, zeolitic imidazolate framework-8 (ZIF-8), a type of MOFs, is an efficient vaccine delivery system [182,183]. Imidazole, the basic structural unit of ZIF-8, is the smallest molecular weight molecule among the TLR agonists and can be modified to produce antagonist molecules or TLR-specific agonists, particularly for TLR-8 and TLR-7 [184,185]. ZIF-8 can be passively primed to the draining lymph node to activate the innate immune response at the injection site [186]. ZIF-8 binds to TLRs in APCs in response to pH degradation that activates the MyD88-dependent pathway, which activates nuclear factor κB, produces type I IFN- β and IL-6, and increases CCR-7 and CD80 expression in APCs responding to the TLR [186]. ZIF enables sustained antigen release (Fig. 5) [187]. However, ZIF-8 is synthesized under harsh conditions and is not tolerated by viral antigens that are sensitive to pH or ionic strength. The key to solving this problem lies in balancing ZIF-8 crystal growth with viral integrity. Wang et al. [188] successfully encapsulated inactivated FMDV in ZIF-8 with high encapsulation efficiency by lowering the pH of 2-methylimidazole solution to 9, adding cetyltrimethylammonium bromide, or increasing the dosage of Zn^2+^ and achieved a substantial increase in its thermal stability by about 5 °C. Upon inoculation, the vaccine significantly increased specific antibody titers and promoted the differentiation of memory T cells. MOFs have been investigated in the development of veterinary nanovaccines against both viral and bacterial infections. For example, Ding et al. [189] developed a nanovaccine using MOFs as a nanovaccine delivery system (Cap@ZIF-8-CpG) encapsulated with porcine circovirus type 2 antigen (Cap) and CpG immune enhancers. The vaccine induced a potent humoral immune response, resulting in a considerable increase in IgG antibody titers and increased cytokine secretion. Hu et al. [190] prepared a nanovaccine based on the encapsulation of the outer membrane phosphoporin of *Klebsiella pneumoniae* within ZIF-8, which induced significantly higher IgG antibody titers, a higher splenocyte proliferation index, and increased cytokine levels after subcutaneous immunization. This nanovaccine was comparable in preventive efficacy to a vaccine formulated using Freund’s adjuvant.

Overall, MOFs show great potential for use as vaccine adjuvants, protecting and delivering antigens, and overcoming the cold chain. To enhance the efficiency of MOFs in delivering antigens, it is crucial to optimize their composition and structure, enabling the introduction of a wide range of organic ligands. The potential toxicity of MOFs poses a major obstacle to their clinical translation, necessitating sustained attention and in-depth, systematic research.

When developing veterinary vaccines using nanomaterials, we typically target the nanomaterials to address the deficiencies of different types of vaccines based on the challenges they face in their functionality. Inactivated and subunit vaccines are weakly immunogenic and require multiple inoculations. Nanomaterials with multivalency, such as SAPN, are selected to exhibit multiple antigenic epitopes in high density to enhance their immunogenicity and also protect subunit vaccines from enzymatic degradation; live attenuated vaccines have potential risk of virulence regression and poor stability, so materials with high stability, such as MOFs, are selected to improve their stability and reduce the risk of virulence regression; enzymes quickly degrade mRNA vaccines and DNA vaccines, and materials such as LNPs and PLGA are selected to protect nucleic acids, as well as to enhance interaction with cell membranes and improve intracellular transport of DNA vaccines. Then, according to the nature of the selected materials and the vaccine route, other nanomaterials are introduced or modified to perfect their targeting and controlled release ability, further improving the effectiveness and safety of the vaccine. Finally, we summarized the properties, advantages and disadvantages, and optimization strategies of various nanomaterials for veterinary vaccine development (Table).

**Table.** Properties, advantages, disadvantages, and optimization strategies of nanomaterials for veterinary vaccine development

Categories	Nanomaterials	Properties	Advantage	Disadvantage	Optimization strategy
Biomimetic nanomaterials	VLPs	Safety, heterogeneity, highly ordered structural organization, and high immunogenicity	Polyvalency, self-adjuvant, targeted localization, and stimulation of immune response	Dependent on cold chain storage and transportation and unstable after modification	Surface functionalization or coating and biomineralization
	CNPs	Biocompatible, low biotoxicity and immunogenicity, and preservation of functional cell membrane components	Prolonged vivo circulation time and half-life targeted	Simple function of a single-cell membrane	Development of hybrid membranes
	EVs	Biocompatibility, safety, natural origin and composition, and bioinformatic capacity	Self-adjuvant and unique targeting and functional properties after functional modification	Complex surface modification, poor encapsulation capacity, and low yield	Engineering
Polymeric nanomaterials	CS	Biocompatibility, safety, low toxicity, biodegradability, mucosal adhesion, and ease of modification	Targeted, applicable to multiple routes of administration, and activates numerous signaling pathways	Poor solubility	Introduction of hydrophilic groups and grafted polymers
	PLGA	Biocompatible, biodegradable, and in vivo degradation, nontoxic, and low immunogenicity	Increases antigen stability and releases the antigen	Negatively charged surface, poor mucosal adhesion, and immune-enhancing ability	Binding to cations by covalent coupling or surface physical adsorption and hydrophilic modifications
	PEI	Strong positive charge, pH buffering capacity, good water solubility, and easy to synthesize	Nucleic acid transfection agents and development of positively charged nanomaterials	Toxicity and weak specific recognition	Modifications, linked oligosaccharides or fluorescent labeling, etc.
	NG	Biocompatible, customizable size, hydrophilic, high specific surface area	Polyvalent, response to various stimuli, and multiple routes of administration	Possible poor stability	Surface modification
	Dendrimer	Size adjustable, low immunogenicity, and highly water soluble, with many modifiable terminal groups	Self-adjuvant, multivalent; nature and properties can be adjusted while reducing toxicity	Overall, positively charged dendrimers may cause cytotoxicity	Anionic dendrimers are generally nontoxic
LNPs		Biocompatibility and safety	Widely used for nucleic acid package protection and delivery and some with adjuvant activity	R&D relies on discovery-driven and low in vivo screening throughput	Design of ionizable lipids, surface-modified LNPs, and high-throughput screening
Self-assembling protein NPs		Biocompatibility, biodegradability, safety, and highly organized structure	Self-adjuvant and multivalent	Low stability	Addition of stabilizers or protectants and chemical cross-linking
Other nanomaterials	MeNPs	Biocompatibility, optical and electrical properties, and stability	Immunostimulatory molecules, delivery system, and antimicrobial effect	Potential toxicity	Surface modification
	CNTs	Stability, osmotic and adsorption capacity, low toxicity, and immunogenicity	Protects the antigen and connects many ligands	Hydrophobicity	Surface functionalization
	MOFs	High specific surface area, customizable dimensions and forms, and easy to prepare and modify	Improving vaccine stability and overcoming the cold chain	Unstable in aqueous media	Selection of suitable metal–ligands and postmodification methods to modulate properties

## Application of Nanomaterials in Veterinary Vaccine Development

Although vaccination is an effective method of controlling infectious diseases, there are still multiple diseases for which there are no effective vaccines [191]. Nanomaterial-based vaccines can address these challenges by optimizing vaccine development and scaling up manufacturing. The following is an overview of nanovaccines developed in recent years, designed to enhance immune responses and improve overall vaccine safety and efficacy.

### Application of nanomaterials to the development of antiviral veterinary vaccines

#### Foot-and-mouth disease

FMD is a transboundary pathogen that can infect more than 70 species of even-toed ungulates, with 7 serotypes and over 100 subtypes. FMD spreads efficiently and is still endemic in large parts of the world [192,193]. An inactivated vaccine is the primary strategy for preventing and controlling infections with FMDV. Still, this vaccine requires a high containment facility for handling the live virus, and the duration of immunity is short, which does not allow for effective pathogen eradication [194]. VLPs not only overcome the limitations of current vaccines but also can differentiate between infected and vaccinated animals. Aparna et al. [195] designed a vaccine against FMD Asia-1 serotype VLPs to protect guinea pigs with 85.6% efficacy, which could serve as an alternative to conventional vaccines. However, the antigen dose needs to be optimized for better protection, and the expression of VLPs needs to be increased to achieve a higher yield, making it more capable of meeting actual production demands. In contrast, Gao et al. [196] wrapped DC membranes containing FMDV antigenic information on the surface of PLGA NPs encapsulating IL-2 to prepare a biomimetic NP vaccine (Biom@DC) for the treatment of FMD, which led to the activation and proliferation of T cells and significantly lowered the proportion of suppressor regulatory T cells. This design scheme drastically optimized antigen delivery, highlighting the importance of the synergistic effects of multiple components in vaccines. The introduction of numerous functional components can also be considered in the development of other vaccines to achieve complementary advantages and enhance the overall performance of the vaccine. However, the vaccine preparation process is expensive and complex, and the effectiveness has not been validated in animals, so it still needs to be optimized. In addition, nanoemulsions have also been used to develop FMD vaccines. Miao et al. [197] demonstrated that mice vaccinated with FMD vaccines prepared using a double-emulsified adjuvant containing ginsenoside Rh2 had significantly higher neutralizing antibody (NAb) titers and splenocyte proliferation rates than mice immunized with FMD vaccines prepared with a double-emulsified adjuvant alone. However, saponins have hemolytic properties and require continued attention in development and application.

#### Porcine epidemic diarrhea

Porcine epidemic diarrhea (PED) causes acute diarrhea, vomiting, and dehydration in newborn piglets with high mortality [198]. Current commercial PED vaccines have poor cross-protection and are not effective against mutated and evolved virus strains [198,199]. How to use nanomaterials to design vaccines against emerging viral strains in a shorter period is a question worth exploring. Yang et al. [200] utilized 3 different kinds of self-assembled NPs with S1 protein’s receptor-binding C-terminal structural domain (CTD) and N-terminal structural domain (NTD) as targets, designated as CTDnps, NTDnps, and NTD/CTDnps. Different ratios of NTD/CTDnps induced significantly higher NAb titers compared to NTDnps and CTDnps alone. When the ratio of CTDnps and NTDnps is 1:3, the protection rates and induced NAb titers in piglets are as high as 83.33% and 92.92%, respectively, compared to the commercially available vaccine. This suggests that combining CTD and NTD antigens enhances the efficacy of nanovaccines against the PED virus (PEDV). More importantly, the nanovaccine design is based on the covalent linkage strategy of the SpyTag/SpyCatcher system, which can rapidly adjust the vaccine antigens against the constantly mutating strains and can be used against different strains. The concept of rapid antigen replacement is worth learning and applying to the development of vaccines for other mutable viruses. In addition, the mRNA vaccine platform can rapidly update immunogens. It has the potential to be broad spectrum in response to emerging PEDV strains, meeting the need to cope with viral variation. Zhao et al. [201] demonstrated that LNP-encapsulated mRNA encoding full-length PEDV spiking protein (S) vaccine (S mRNA-LNP) induced a potent PEDV-specific immune response in vivo and protected piglets from PEDV infection. Most importantly, S mRNA-LNP also adequately immunizes newborn piglets in colostrum after sow immunization. However, the cross-neutralization ability of this vaccine against different genotypes of PEDV is insufficient to counteract infections with multiple genotypes of PEDV effectively, and further studies are still needed.

#### African swine fever

African swine fever (ASF) is an infectious, acute, febrile illness triggered by the ASF virus (ASFV), with a lethality rate of nearly 100% in pigs. There is still no safe and efficacious vaccine for ASF [202]. mRNA vaccines, which offer high safety, effectiveness, and cost-effectiveness, are a compelling choice for developing an ASF vaccine. Gong et al. [203] constructed an ASF p30 mRNA vaccine (mRNA/Man-LNP) utilizing mannose-modified LNPs, which induced a powerful IgG titer and stimulated both CD4 and CD8 T cells. However, a single antigen may not provide sufficient protection. Delivery systems capable of delivering multiple antigens, such as SAPNs, are multivalent and are an ideal alternative. Sun et al. [204] conjugated antigens to multiple T cell epitopes (TEPs) of ASFV via the SpyCatcher/SpyTag system and displayed the self-assembled NPs, thus constructing nanovaccines (TEP-Spy-NPs). TEP-Spy-NPs produced higher TEP-specific antibody titers and higher numbers of splenic lymphocytes than TEP-alone immunization after the second booster immunization, inducing robust cellular and humoral immunity more intense than TEP-alone ones; meanwhile, Song et al. [205] constructed a self-assembled nano-ASFV vaccine (NanoFVax), targeting DCs by covalently coupling the predominant T cell and B cell epitopes of highly immunogenic ASFV antigens with self-assembled ferritin and fusing it with the chemokine receptor X-C motif chemokine ligand 1. Compared to monomeric proteins, NanoFVax induces stronger T cell responses, with high levels of antibody responses against ASF that last more than 231 d. This demonstrates the great potential of SAPN in developing ASF vaccines, which can be combined with specific receptors on the surface of target cells to optimize delivery and enhance the effectiveness of the vaccine.

#### Pseudorabies

Pseudorabies virus (PRV) can affect many domestic and wild animals [206]. Live attenuated or inactivated vaccines are the most efficacious means of preventing and treating PR. Still, with the emergence of PRV variants, the effectiveness of existing vaccines has been drastically reduced [207]. Therefore, the development of new vaccines against PR is urgently needed. As described above, MOFs are nanomaterials with great potential, as they are customizable in terms of size and shape and can be easily prepared and modified. However, the poor stability of MOFs in aqueous media severely limits their practical applications in vaccine development. Fortunately, this deficiency can be mitigated by selecting suitable metal ligands and incorporating surfactants or hydrophilic polymers onto the surface of MOFs. The following 2 studies utilized different MOFs as potential vaccine adjuvants. Liao et al. [208] adopted the polyacrylic-acid-modified Carbopol dispersed zirconium-based MOF UIO-66 (U@PAA-Car) as an adjuvant for PR vaccines, which, compared to the commercial adjuvants Carbopol or U@PAA, induced higher splenic cell proliferation and cytokine secretion, IgG2a/IgG1 ratio, specific antibody titers, and provided higher protection rates in mice and pigs. Yin et al. [209] constructed an inactivated PRV vaccine using alginate-dialdehyde-coated aminated ZIF-7/8 NPs (ZIF-7/8-ADA NPs), which accelerated antigen presentation, enhanced T_H_1/T_H_2 immune responses, and achieved a preventive effect similar to that of the commercial ISA201 that is superior to alum. This demonstrates MOFs’ great potential and broad prospects in developing novel PRV vaccines and provides valuable ideas and experience for developing subsequent vaccines. By combining the advantages of different nanomaterials to fully utilize the properties of various nanomaterials, composites with better overall performance can be created to meet the actual needs of vaccine development.

### Application of nanomaterials in the development of antimicrobial veterinary vaccines

#### Bordetella bronchiseptica

Bordetellosis is a respiratory disease triggered by *Bordetella bronchiseptica* (Bb) infection, which is widely spread, hard to cure, and constitutes a menace to mammals such as rabbits and pigs, as well as immunocompromised humans [210,211]. Traditional vaccines against Bb can elicit an adequate antibody response, but the protective effect is limited.

Therefore, novel, safe, and effective vaccines must be developed to prevent Bb. Outer MVs (OMVs), a type of EVs, are released during the growth of Gram-negative bacteria and hold a high quantity of PAMPs, an effective antigenic candidate [212,213]. However, OMV instability and heterogeneity seriously affect its immune efficacy, and the combination with NPs can strengthen the stability of OMV [212,214–216]. Huang et al. [217] coated OMV onto polyethylene-glycolated nano-*Rehmannia glutinosa* polysaccharide (pRL) to develop a nanovaccine (pRL-OMV). pRL-OMV remarkably increased DCs’ proliferation and maturation, as well as cytokine secretion, and exhibited excellent LNs targeting, facilitating the generation of bacterial-specific antibody responses and potent mixed cellular responses against Bb infection. In addition, Li et al. [218] prepared a stable OMV vaccine (CNP-OMV) using OMV-coated CS NPs. CNP-OMV significantly promoted cell proliferation and cytokine secretion, produced high levels of IgG, and induced a mixed immune response in rabbits T_H_1/T_H_2/T_H_17. Meanwhile, CNP-OMV significantly reduced bacterial invasion in the lungs of the attacking rabbits, exhibiting a protective effect on the lungs. These studies provide a scientific basis and new ideas for the advancement of novel and effective Bb vaccines, highlighting the great potential of OMV conjugated with NPs for antibacterial vaccine development.

#### *Mycobacterium avium* subspecies paratuberculosis

*M. avium* subspecies paratuberculosis (MAP) triggers paratuberculosis or Johne’s disease, which affects the gastrointestinal health of ruminants, causing persistent diarrhea, decreased productivity, and wasting, resulting in economic losses [219,220]. It may be linked to Crohn’s disease, which poses a threat to human health [221,222]. Mucosal vaccines are more effective than injections in efficiently preventing infections caused by mucosal pathogens, and polysaccharide modification and NP encapsulation are essential strategies for addressing gastrointestinal challenges. Liu et al. [223] developed a PLGA-based ternary polyelectrolyte complex (PEC) to deliver MAP fusion antigenic protein (HBf). Oral administration to mice decreased bacterial load and liver pathology, enhanced splenic T cell responses, and promoted the secretion of intestinal mucosal IgA and specific antibodies. However, PEC can only be stored at 4 °C for 7 d, which severely limits its practical application. In another study, the same MAP antigen HBf was selected. Liu et al. [224] utilized PLGA to encapsulate all-trans retinoic acid and formed a “nanocoat” with polydopamine to adsorb the TLR9 agonist CpG and antigen, generating pathogen-mimicking NPs (PLPCa NPs). After intramuscular injection, the PLPCa NPs formed an immune-rich microenvironment at the injection site, which enhanced the whole-body immune response and induced potent IgA levels, significantly decreasing bacterial burden and inflammation in the intestinal tract. This study highlights the importance of designing targeted pathogen-mimicking delivery systems tailored to the characteristics of different pathogens in the development of vaccines to enhance their immunogenicity

Overall, whether antiviral or antibacterial, nanomaterials have shown great potential and are increasingly used in veterinary vaccine development. Because of the variety of nanomaterials and the further development of nanotechnology, not only the immunization effect of vaccines can be enhanced, but also the stability of vaccines can be improved by selecting suitable nanomaterials according to the limitations of existing veterinary vaccines and the characteristics of pathogens. Moreover, a variety of nanovaccines have been studied for the same infectious disease for selection. In the development of veterinary vaccines, optimizing the convenience of veterinary vaccination while ensuring the efficacy of veterinary vaccines and low-cost scale-up of production are the top priorities. On this basis, it would be even more perfect if a single vaccination could be realized to provide long-term protection. In the future, with the advancement of nanotechnology and its multidisciplinary applications, nanomaterials are expected to enhance the development of veterinary vaccines, promoting efficiency and economy.

## Conclusion and Outlook

Nanomaterials have demonstrated remarkable potential in developing veterinary vaccines to prevent zoonotic diseases. Under their nanoscale size and high specific surface area, nanomaterials can enhance the immunogenicity and stability of antigens, improve the bioavailability of antigens, and promote the cross-presentation of antigens, thereby modulating immune responses. At the same time, nanomaterials also extend the vaccination route, which can be administered orally, intranasally, or through immersion, among other routes, and activate both mucosal and systemic immunity. This significantly reduces the manpower cost of large-scale immunization and makes veterinary vaccination more convenient and practical. In addition, nanomaterials can further enhance the targeting of specific cells or tissues after modification, effectively control the release process of antigens, reduce the impact on nontarget sites, and improve the immune effect while minimizing adverse reactions, thereby prolonging the duration of the antigen’s role. Most importantly, nanomaterials such as MOFs have the potential to break through the limitations of traditional transport and storage and overcome cold chain storage; SAPNs, dendrimers, and NGs are multivalent, allowing the development of veterinary vaccines against a wide range of homologous and heterologous pathogens, and have the potential to design single-dose vaccines that can achieve long-term memory without booster injections, which is highly significant for improving the ability of prevention and control of animal diseases and guaranteeing stable development of the animal husbandry industry. With its modular design, rapid response, and flexible adaptation, the plug-and-play platform can significantly shorten the research and development cycle, offering great potential in the face of sudden pandemic diseases.

However, although approved veterinary nanovaccines are on the market, nanomaterials still face challenges in developing veterinary vaccines. First, safety is an ongoing concern. Some nanomaterials, such as PEIs, NGs, and dendrimers, are potentially toxic. Although the toxicity will be reduced after modification, an in-depth understanding of the internal distribution of nanomaterials in the animal body is still needed, as well as the establishment of a standardized evaluation system of nanomaterials for veterinary vaccine development, and the clarification of the thresholds for the correlation between the particle size, the electric charge, and other physical properties and the safety, which is a key prerequisite for the development of veterinary vaccines using nanomaterials. Second, vaccines based on nanomaterials such as EVs and CS may be costly, difficult to purify, and complicated to produce, posing challenges for clinical translation. Future research should aim to optimize the production process of nanomaterials to improve stability and consistency in large-scale production, enhance their tolerance to temperature fluctuations, and reduce reliance on the cold chain, which could be beneficial for expanding the application of nanomaterials in veterinary vaccines. In addition, nanomaterials are primarily used in combination with multiple nanomaterials or with modified nanomaterials in the development of veterinary vaccines, and screening out the best nanomaterial combinations among many nanomaterials is essential but complex. In the future, we can establish a substantial virtual library of nanomaterials and ligands to simulate the entire process of in vivo delivery and immune activation in animals, screen the optimal vaccine formulation, and then verify its effectiveness through experimental evaluation, which is an effective way to address this challenge. Ultimately, transitioning nanovaccines from small-scale laboratory synthesis to commercial production is a challenging endeavor. Fortunately, with the development of nanotechnology, it is possible to optimize the production process and reduce production costs, thereby accelerating its translation from the laboratory to the clinic.

Although many unresolved issues remain to be explored in nanovaccines, the mechanisms by which nanomaterials function as vaccine adjuvants will be further elucidated as our understanding of nanomaterials continues to grow. Through rational development and continuous multidisciplinary cross-exploration for improvement, nanomaterials are expected to achieve more effective immune activation and broader pathogen coverage while addressing the challenges facing current vaccines. Given the rapid development of nanovaccines, the prospect of low-cost and high-quality nanovaccines in veterinary medicine is very bright, and it is believed that the life and health of veterinary species can be improved through them shortly.
